# Opportunistic screening for atrial fibrillation in a real-life setting in general practice in Denmark—The Atrial Fibrillation Found On Routine Detection (AFFORD) non-interventional study

**DOI:** 10.1371/journal.pone.0188086

**Published:** 2017-11-13

**Authors:** Jonas Hald, Peter Bo Poulsen, Ina Qvist, Lisbeth Holm, Dorte Wedell-Wedellsborg, Lars Dybro, Lars Frost

**Affiliations:** 1 Lægerne Odingården (GP-clinic), Viborg, Denmark; 2 Pfizer Denmark, Health & Value, Ballerup, Denmark; 3 Department of Cardiology, University Research Clinic for Innovative Patient Pathways, Regional Hospital of Silkeborg, Silkeborg, Denmark; 4 Pfizer Denmark, Internal Medicine, Ballerup, Denmark; 5 Department of Clinical Medicine, Aarhus University Hospital, Aarhus, Denmark; Mayo Clinic, UNITED STATES

## Abstract

Atrial fibrillation (AF) is a chronic disease with an incidence increasing steeply by age and affecting more than 11 million patients in Europe and the United States. Diagnosing AF is essential for the prevention of stroke by oral anticoagulation. Opportunistic screening for AF in patients ≥65 years of age is recommended by the European and Danish Societies of Cardiology. The study aim was to examine the detection rate of AF in consecutively screened patients in the primary care setting in Denmark. In an open, non-interventional, cluster, multicenter, cross-sectional, observational study patients ≥65 years of age entering consecutively into general practice clinics were invited to nurse-assisted opportunistic screening for AF. The General Practice (GP) clinics participating were randomized to patient inclusion in three age groups: 65–74, 75–84, and ≥85 years respectively. All patients underwent pulse palpation followed by 12-led electrocardiogram in case of irregular pulse. Two cardiologists validated all electrocardiogram examinations. Forty-nine general practice clinics recruited in total 970 patients split into three age groups; 480 patients (65–74 years), 372 (75–84 years), and 118 patients ≥85 years of age. Co-morbidities increased by age with hypertension being most frequent. Eighty-seven patients (9%) were detected with an irregular pulse, representing 4.4%, 10.5% and 22.9%, respectively in the three age groups. Assessment of electrocardiograms by the GP showed suspicion of AF in 13 patients with final verification of electrocardiograms by cardiologists revealing 10 AF-patients. The highest detection rate of AF was found in the ≥85 age group (3.39%) followed by the 65–74 age group (0.83%) and the 75–84 age group (0.54%). Opportunistic screening of AF in primary care is feasible and do result in the detection of new AF-patients. Close collaboration with cardiologists is advisable to avoid false positive screening results.

## Introduction

Atrial fibrillation (AF) is a supraventricular tachyarrhythmia characterized by uncoordinated atrial depolarizations and consequently disrupted atrial function [1,2]. AF is a chronic disease with an incidence increasing by age and the prevalence has been reported to rise from 2.3% in those over 40 years of age to around 8% in those over 80 years [3] to as high as 17% in those aged 85 years and above [4,5,6]. In the developed world the prevalence is approximately 1.5–2% of the general population with more than 6 million people in Europe and 5 million people in the United States currently suffering from AF [1,2,7]. Projections suggest these figures to at least double in Europe and USA by 2050 due to an aging population [1,2,8]. In Denmark, 312,420 patients were reported from Danish hospitals to the National Patient Registry having a first-time AF diagnosis from 1983 to 2012 with a population incidence of AF reported as of 1.4% per year on average in the period 2000 to 2012 [9].

Treatment of AF and stroke prophylaxis with oral anticoagulation is important as undiagnosed AF carries risk of ischemic stroke as potential serious clinical outcomes, including risk of death [1,10]. Hence, diagnosing AF is considered as a prerequisite for risk management [11]. Today diagnosing AF in routine daily clinical practice is based on the presence of symptoms such as dyspnea, chest pain, palpitations, etc. or AF-induced sequelae such as presence of stroke, peripheral arterial thromboembolism, or heart failure. However, one third of all AF-patients does not display any symptoms [4] and are therefore not easily detectable and likewise most often left undiagnosed. It has been shown that pulse palpation detecting an irregular pulse may indicate AF. In case of irregular pulse a follow-up verification with an electrocardiogram (ECG) must be performed to confirm or exclude the presence of AF. Detection and diagnosing of AF can be made by several, including general practitioners (GP’s), specialists or by health care professionals during inpatient stay at the hospital.

In order to prevent detrimental outcomes of AF such as stroke, systemic embolism, heart failure, or death and to be able to detect the large share of undiagnosed AF-patients today as well as counter the future projected growth in the prevalence of AF, the guidelines from the European Society of Cardiology (ESC) (2010, 2012 update, and 2016) recommend (Class 1) opportunistic screening of AF in patients ≥65 years of age using pulse palpation, followed by an ECG when the pulse is irregular [1,2,11]. This guideline recommendation on opportunistic screening of AF is based on a single randomized controlled trial from the United Kingdom by Fitzmaurice et al. [12]. Recently, opportunistic (single time point) screening was also recommended in a report by the AF-SCREEN International Collaboration and in the European Heart Rhythm Association (EHRA) consensus document [13–14], as well as found likely to be cost-effective in the GP-setting [14–15]. According to the guidelines opportunistic screening should be adopted in primary care and be performed by the GPs or the clinic nurse. The ESC guidelines have been endorsed by the Danish Society of Cardiology and made available to the Danish GPs [16]. To secure repeated screening for AF, the Danish national AF guidelines recommend pulse palpation at any contact to the health care system in people aged 65 year or above.

The aim of the present study was based on the ESC guideline recommendation on opportunistic screening to investigate the detection rate (undiscovered prevalence) of newly diagnosed AF patients among consecutively screened patients in routine daily clinical practice in Denmark. The patients entered in the opportunistic screening study were stratified into different age groups: 65–74 years, 75–84 years, and ≥85 years, respectively.

## Materials and methods

The Danish health care system is predominantly tax-financed, and all citizens have free and equal access to health care. The study was designed as an open, non-interventional, cluster, multicenter, cross-sectional, observational study in the primary care setting in Denmark. The study was descriptive and explorative and designed as a one-arm cross-sectional study of single time-point opportunistic screening the day the patient, for any reason, turned up at the clinic for a consultation. As this was a one all screening activity, follow-up was not part of the study purpose. Patient inclusion was consecutive, meaning that every relevant patient (i.e. fulfilling the study inclusion criteria–see below) was asked for participation during the inclusion period from January to March 2016, and, in case of consent, included in the study.

### Cluster randomization at clinic level

Focusing on the ≥65 years patients, as recommended by the cardiology guidelines, and due to their by age increasing risk of having AF, the patients included in the study were stratified for screening into three different age groups: 65–74 years, 75–84 years, and ≥85 years. There was no randomization among patients in the study, because this is against the non-interventional premise of the study to follow routine daily clinical practice. Instead cluster randomization was adopted, and consequently the GP clinics participating were randomly allocated to one of the three age groups. GP investigators working together in group practice clinics were allocated to the same age group of study patients.

### Inclusion criteria

As in the study by Fitzmaurice et al. [12] as well as other systematic screening studies the present study aimed at nurse-assisted screening sessions. Consequently the GP investigators participating in the study collaborated with the clinic nurse and were jointly responsible for patient inclusion as well as for pulse palpations. Furthermore, all investigators were required to have access to an ECG recorder in the clinic. The expected number of investigators in the study was 60 GPs, including clinic nurses.

Patients were consecutively asked for their consent to participate, when they consulted the clinic. To be included in the study patients had to fulfill all of the following inclusion criteria’s: 1) patients were not previously diagnosed with AF as confirmed by record examination, 2) patient self-admission to the general practitioner was for any reason (opportunistic), and not due to the screening activity itself, 3) patients in scope had to belong to the specific age groups that the clinic was randomized to in the study, and 4) patients had to personally sign and date the informed consent document indicating that they had received information on all pertinent aspects of the study.

### Study size

Due to the study design—one-arm cross sectional study without any control arm, no follow-up, and no delta value (difference) revealed, traditional sample size (power) calculation was not feasible. Instead information from sample sizes in historical trials were examined [12,17–27]. Furthermore, analysis of the minimum sample sizes for the three different target groups needed were calculated using a formula for sample size calculations in prevalence studies by Daniel [28] and a corresponding calculation by Naing et al. [29]. This is based on information on the prevalence rate of AF in Denmark [30], data on number of Danes in the different age groups [31] and a level of confidence of 95%. The prevalence rate of AF among all Danes were in 2011 8% among the Danes 65+ years Danes and split into 5.5% for the 65–74 years, 10% for the 75–84 years and 15% for the 85+ years [30]. These sample size calculations showed a minimum of 257 patients (precision of 0.028) in the 65–74 year age group, a minimum of 140 patients (precision of 0.05) in the 75–84 year age group and a minimum of 193 patients (precision of 0.05) in the 85+ year age group.

Looking into the share of patients in the specific age group in relation to the total number of 65+ years of age citizens in Denmark it was decided to double the number of patients required in the 65–74 year age group to a target of 500 patients recruited by 25 clinics. Similarly with the 75–84 year age group it was decided to nearly triple this compared to the calculation looking at its share, why the target in this group was set to 400 patients recruited by 20 clinics. Finally, because 85+ citizens in Denmark only accounts for 11% of the 65+ years of age population it was decided only to increase the target in the 85+ age-subset to 300 patients to be recruited by 15 clinics. For each investigator this would require a recruitment target of 20 patients. In total, the present study aimed to include 1,200 patients ≥65 years of age with a reasonable margin to the minimum sample size targets.

### Study endpoints

The primary study endpoint was: the percentage of newly detected AF-patients in each of the three age groups. Secondary study endpoints were: patient characteristics in terms of socio-demographic and clinical characters, thromboembolic risk level and AF risk profile of all screened patients irrespective of the presence or absence of AF. The thromboembolic risk level was calculated using the CHA_2_DS_2_-VASc prediction score, where 1 point corresponds to an annual risk of stroke of 2.8% and indicates that anticoagulation therapy should be considered [2]. The CHA_2_DS_2_-VASc scoring system includes the following characteristics: Congestive Heart failure (C), hypertension (H), Age ≥75 (doubled) (A), Diabetes (D), Stroke (doubled) (S), Vascular disease (V), Age 65–74 (A), and Sex category (female) (S).

### Screening procedure

The opportunistic screening procedure was in accordance with the Danish Society of Cardiology endorsed ESC guidelines [1,2,11,16]. Screening was initiated by obtaining informed consent, preceding pulse palpation of the patient during a routine medical consultation, and followed by recording of ECG, if an irregular pulse was detected, to verify diagnosis of AF [1,2]. Similar definitions are found in previous studies and in a Cochrane Review [3,12,23]. The delegated clinic nurse measured the included patients radial pulse by palpation with the second, third and fourth fingers. The pulse beats were counted and interpreted by the nurse in a defined period, e.g. 30 or 60 seconds (rate per minute). In case of an irregular pulse the patient underwent an ECG examination performed by the GP or by the delegated nurse. The ECG performed was a 12-lead ECG. An ECG recording was performed for all patients being detected with an irregular pulse. The ECG recordings were collected post study for blinded specialist examination performed by two skilled AF specialists. One specialist belonging to the study steering committee examined the ECG tracings as received from the investigators and in parallel a specialist from a Regional hospital examined printed copies. The two specialists were without knowledge on the ECG interpretations made by the GP investigators and were also blinded to one another.

Based on the result of the opportunistic screening and the CHA_2_DS_2_-VASc score calculated stroke risk, further actions like e.g. specialist involvement, additional tests and treatment considerations were expected to be initiated by the GP in terms of the individual patient. This was, due to the non-interventional design, however, not part of or the aim of the present study, and therefore not recorded.

### Data recorded

Study data for each participating patient were entered by the GP or the delegated clinic nurse into an electronic Case Report Form (eCRF) designed for the study. Information on inclusion criteria, specifically confirmation of no previous AF diagnosis by record examination, socio-demographic variables; age, gender, height and weight (both used for the calculation of Body Mass Index (BMI)), last visit by the patient in the clinic for any reason, as well as region and city of the clinic were collected. In line with the ESC guidelines [1,2] a number of historic or specific clinical symptoms/diseases to describe a patient’s AF risk profile were also recorded for each participant and based on information already available in the patients’ medical records, including history of: *hypertension* (defined by systolic blood pressure above 140 mmHg and/or the patient is already on treatment), *symptomatic heart failure*, *diabetes mellitus*, *chronic obstructive pulmonary disease (COPD)*, *known kidney function* (defined by the existence of a laboratory test (creatinine/eGFR) within the last year), *valvular heart disease* (defined by the presence of mechanical heart valve), *coronary artery disease* (defined as angina pectoris, previous MI or by-pass), *sleep apnoea* (reported by the patient or family members as experienced), *obesity* (defined as a BMI ≥ 30 kg/m^2^). All diseases and symptoms were to be recorded if known by the clinic nurse or the GP, as no additional testing or diagnostic procedures were initiated as part of the study. Information on the dependent variables, the *pulse rate* of the patient, as well as the *ECG result in terms of signs of AF* in case of the detection of an irregular pulse were also recorded by the clinic nurse or GP in the eCRF. The risk of stroke was calculated using the CHA_2_DS_2_-VASc stroke risk prediction score for all patients included. During the study period, a score of ≥1 point using the CHA_2_DS_2_-VASc prediction tool corresponds to an annual risk of stroke indicating anticoagulation therapy should be considered [2].

### Statistical analysis

The data to be analyzed were the entire sets defined as all patients entered the study. Continuous endpoints were summarized using descriptive statistics (n, mean, standard deviation, minimum, median and maximum, 95% CI, and number of missing data). Similarly, discrete endpoints were summarized in frequencies and percentages (95% CI and number of missing data). All confidence intervals were presented from a 0.05 significance level (two-sided).

### Data protection and ethics committee

The study was approved by the Danish Data Agency. Furthermore, the Danish National Board of Health and the National Committee on Health Research Ethics both confirmed that the study was a non-interventional observational study. According to the Danish Medicines Act, the obligation to apply for authorization does not apply to non-interventional studies; hence, approvals by either of these bodies were not required.

## Results

**Fig 1:**
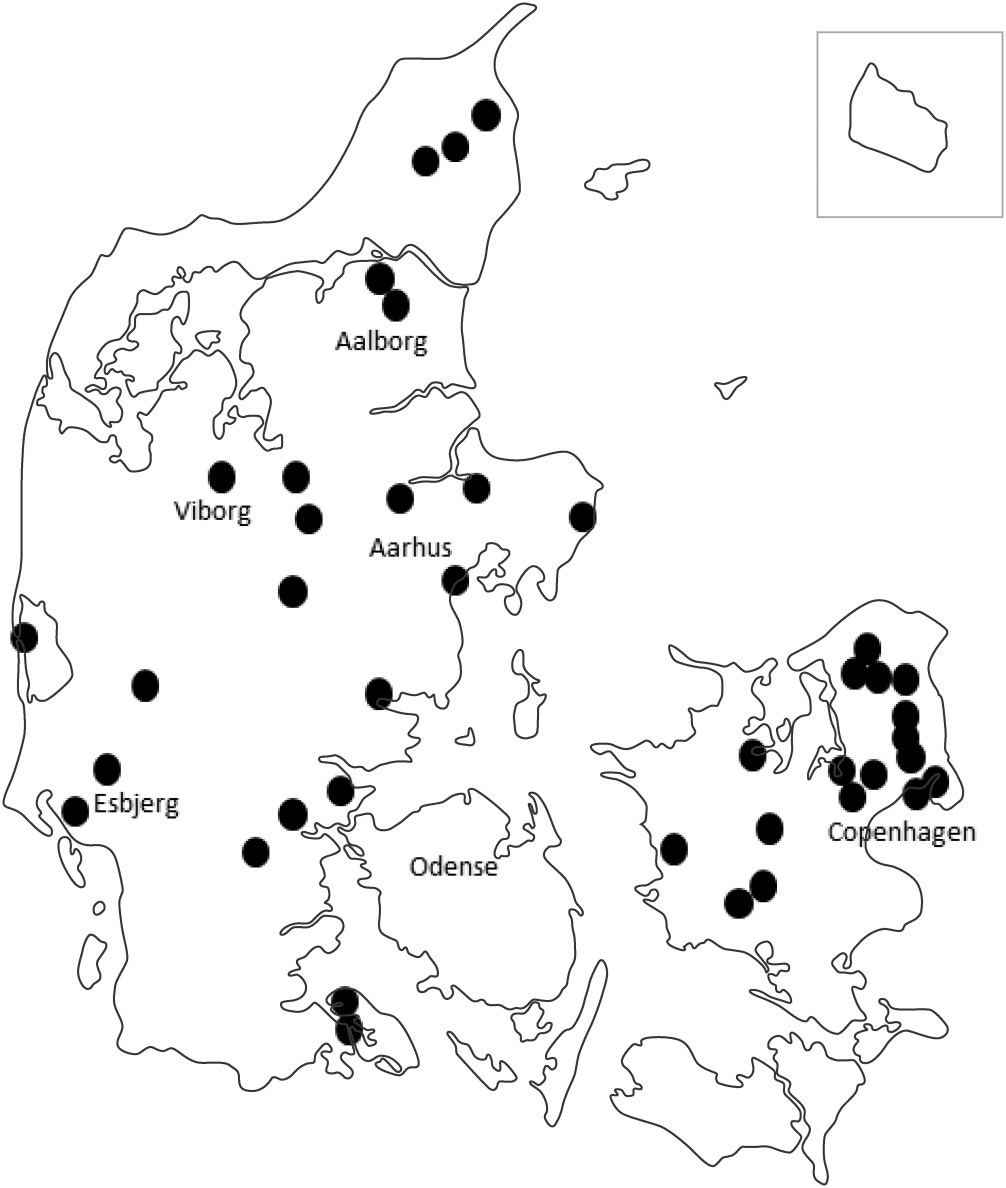
Location of the 49 participating GP-clinics in Denmark.

Although 60 GP clinics fulfilling all criteria’s were recruited and each randomly allocated to one of the three age segments (Table 1), only 49 GP clinics (82%) managed to include patients in the specific age segments. These clinics were geographically located throughout Denmark (Fig 1).

**Table 1:** Subjects screened for AF by opportunistic screening (N = 970).

		Subjects expected	Actual (N)*	Fulfillment (%)**
Number of subjects screened		1.200	970	81
Age group N (%)	65–74	500	480 (49.4)	96
	75–84	400	372 (38.4)	93
	≥85	300	118 (12.2)	39

* N = Total number of subjects included.

** % = Percentage of subjects included compared with subjects expected.

The clinics that did not succeed to include adequate numbers of patients during the screening period, were six clinics randomly allocated to include patients in the oldest age segment, three clinics of the 65–74 age segment and two clinics of the 75–84 age segment.

In total 970 patients ≥65 years of age were included in the study with a split into the three age groups as presented in Table 1 below.

Of all patients slightly more women than men were included (55%); the share of females being markedly increased (71%) in the oldest age group (Table 2). The mean age of the patient-population was 75 years. The patient’s Body Mass Index (BMI) decreased slightly with increasing age (Table 2). Almost all patients screened (99%) had visited their GP for other reasons within the last two years.

**Table 2:** Subjects characteristics (N = 970).

Age group		65–74 (N = 480)	75–84 (N = 372)	≥85 (N = 118)	Total (N = 970)
Gender	Females (%)	257 (53.5)	195 (52.4)	84 (71.2)	536 (55.3)
Last GP visit <2 years ago	Yes (%)	477 (99.4)	371 (99.7)	116 (98.3)	964 (99.4)
Age	Mean (SD)	69.2 (2.7)	78.7 (2.8)	88.0 (3.1)	75.1 (7.1)
BMI	Mean (SD)	27.3 (4.8)	26.7 (4.3)	25.3 (4.6)	26.8 (4.6)

N = Number of subjects.

% = Percentage of subjects.

SD = Standard Deviation

### Medical history

It was confirmed by record examination that the patients included were not previously diagnosed with AF. According to the ESC guidelines, several cardiovascular and other clinical characteristics may be coexisting and associated with atrial fibrillation [1,2,11]. As seen in Table 3 the prevalence of co-morbidities increased with age. The most frequent co-morbidities were hypertension (63%), diabetes (20%), COPD (11%) and ischemic heart disease (10%). Kidney function, quantified by either eGFR or serum creatinine was recorded for 831 patients (86%) with eGFR as the predominant method used (average 59%) (Table 3)

**Table 3:** Medical history (N = 970).

Age group		65–74 (N = 480)	75–84 (N = 372)	≥85 (N = 118)	Total (N = 970)
Hypertension, n (%)	Yes	269 (56.0)	263 (70.7)	82 (69.5)	614 (63.3)
	Unknown	7 (1.5)	1 (0.3)	1 (0.8)	9 (0.9)
Heart failure, n (%)	Yes	12 (2.5)	15 (4.0)	10 (8.5)	37 (3.8)
	Unknown	0 (0.0)	3 (0.8)	1 (0.8)	4 (0.4)
Diabetes, n (%)	Yes	103 (21.5)	72 (19.4)	20 (16.9)	195 (20.1)
	Unknown	5 (1.0)	1 (0.3)	0 (0.0)	6 (0.6)
Stroke or transient ischemic attack, n (%)	Yes	24 (5.0)	26 (7.0)	9 (7.6)	59 (6.1)
	Unknown	2 (0.4)	3 (0.8)	1 (0.8)	6 (0.6)
Chronic obstructive pulmonary disease, n (%)	Yes	44 (9.2)	42 (11.3)	17 (14.4)	103 (10.6)
	Unknown	7 (1.5)	1 (0.3)	0 (0.0)	8 (0.8)
Valvular heart disease, n (%)	Yes	4 (0.8)	5 (1.3)	3 (2.5)	12 (1.2)
	Unknown	3 (0.6)	0 (0.0)	1 (0.8)	4 (0.4)
Ischemic heart disease, n (%)	Yes	35 (7.3)	46 (12.4)	19 (16.1)	100 (10.3)
	Unknown	2 (0.4)	2 (0.5)	0 (0.0)	4 (0.4)
Peripheral arterial disease, n (%)	Yes	16 (3.3)	14 (3.8)	6 (5.1)	36 (3.7)
	Unknown	5 (1.0)	3 (0.8)	1 (0.8)	9 (0.9)
Sleep apnea, n (%)	Yes	10 (2.1)	8 (2.2)	0 (0.0)	18 (1.9)
	Unknown	28 (5.8)	35 (9.4)	5 (4.2)	68 (7.0)
Known kidney function, n (%), and value, mean (SD) - eGFR value (mL/min), mean (SD) - Serum creatinine value (μmol/L),	Yes, eGFR	233 (48.5)	243 (65.3)	99 (83.9)	575 (59.3)
	- value	73.7 (13.6)	67.4 (17.4)	60.1 (19.3)	68.7 (17.0)
	Yes, serum creatinine	176 (36.7)	73 (19.6)	7 (5.9)	256 (26.4)
	- value	79.1 (25.2)	87.2 (25.4)	82.7 (35.7)	81.5 (25.7)

N = Number of subjects.

n = Number of subjects belonging to specific subgroups.

% = Percentage of subjects with condition.

SD = Standard Deviation

### Screening results—Pulse palpation and ECG

Eighty-seven (9%) of the 970 patients screened were detected with an irregular pulse (Table 4 and S1 Table). This rate of irregular pulse increased exponentially by increasing age leading to almost one in four of those being 85 years of age or older having an irregular pulse (Table 4).

**Table 4:** Pulse measurement and ECG results at the GP clinic in terms of sign of AF, including specialist ECG verification (N = 970).

Age group			65–74 (N = 480)	75–84 (N = 372)	≥85 (N = 118)	Total (N = 970)
Pulse palpation	Mean rate (SD)		72.5 (11.5)	72.7 (11.3)	73.9 (10.9)	72.8 (11.3)
Pulse palpation findings	Irregular, n (%)		21 (4.4)	39 (10.5)	27 (22.9)	87 (9.0)
	Regular, n (%)		459 (95.6)	333 (89.5)	91 (77.1)	883 (91.0)
ECG result in terms of sign of AF (GP assessment)	N (irregular pulse = ECG)		21	39	27	87
	GP verified	Yes, n (%)	5 (23.8)	4 (10.3)	4 (14.8)	13 (14.9)
		No, n (%)	16 (76.2)	35 (89.7)	23 (85.2)	74 (85.1)
	Specialist verified	Yes, n (%)	4 (19.0)	2 (5.1)	4 (14.8)	10 (11.5)
		No, n (%)	17 (81.0)	37 (94.9)	23 (85.2)	77 (88.5)

N = Number of subjects.

n = Number of subjects belonging to specific subgroups.

% = Percentage of subjects with condition.

SD = Standard Deviation

The share of patients analyzed with a follow-up ECG examination was numerically similar across age groups (11–12%). Among the 87 patients with irregular pulse, ECG recordings interpreted by the GPs resulted in suspicion of AF in 13 patients, i.e. 14.9% (Table 4). The highest share of patients suspected by the GP to have AF following ECG examination was found in the oldest age group with a detection rate of 3.39% (95% CI: 0.12;6.66) (Table 5 and S2 Table).

**Table 5:** Detection rates of new cases of AF stratified by age groups—GP ECG verified AF cases (N = 13).

Age group	65–74 (N = 480)	75–84 (N = 372)	≥85 (N = 118)	Total (N = 970)
Gender (All)	1.04 (5/480) [95% CI: 0.13;1.95]	1.08 (4/372) [95% CI: 0.03;2.12]	3.39 (4/118) [95% CI: 0.12;6.66]	1.34 (13/970) [95% CI: 0.62;2.06]
Females	0.39 (1/257) [95% CI: 0.00;1.15]	0.51 (1/195) [95% CI: 0.00;1.52]	2.38 (2/84) [95% CI: 0.00;5.64]	0.75 (4/536) [95% CI 0.02;1.47]
Males	1.79 (4/223) [95% CI: 0.05;3.54]	1.69 (3/177) [95% CI: 0.00;3.60]	5.88 (2/34)[95% CI: 0.00;13.8]	2.07 (9/434) [95% CI: 0.73;3.41]

N = Number of subjects.

% = Percentage of subjects with condition.

CI = Confidence Interval.

The detection rates for patients under suspicion by the GP to have AF were markedly lower in the two younger age groups.

Final specialist validation of the 87 ECG recordings by the two independent AF specialists, and by that the 13 patients under suspicion by the GPs to have AF, revealed only 10 patients verified to have a proven diagnosis of AF (Table 6). Three (3) GP suspicions and interpretations of the ECG results in patients in the younger age groups were disapproved by the specialists in representing AF (Tables 5 and 6). In the 85+ age group there was a 100% match between the GP assessment and the specialist validation. In terms of the AF detection rate this lead to a specialist verified detection rate of 1.03% (95% CI: 0.40;1.67) for the age groups in total (Table 6), still with the markedly highest detection rate among the 85+ subset of 3.39%. In perspective, this translates into one patient presenting with ECG verified AF per 30 patients ≥85 year old being opportunistically screened compared to one per 120–186 patients in the two younger age groups. The detection rate following opportunistic screening was nearly twice as high for males (1.38%; 95% CI: 0.28–2.48) than for females (0.75%; 95% CI: 0.02–1.47) (Table 6). By that, when screening ≥85 aged men, one patient with AF will be detected per 17 patients screened.

**Table 6:** Detection rates of new cases of AF stratified by age groups–Specialist ECG verified AF cases (N = 10).

Age group	65–74 (N = 480)	75–84 (N = 372)	≥85 (N = 118)	Total (N = 970)
Gender (All)	0.83 (4/480) [95% CI: 0.02;1.65]	0.54 (2/372) [95% CI: 0.00;1.28]	3.39 (4/118) [95% CI: 0.12;6.66]	1.03 (10/970) [95% CI: 0.40;1.67]
Females	0.39 (1/257) [95% CI: 0.00;1.15]	0.51 (1/195) [95% CI: 0.00;1.52]	2.38 (2/84) [95% CI: 0.00;5.64]	0.75 (4/536) [95% CI 0.02;1.47]
Males	1.35 (3/223) [95% CI: 0.00;2.86]	0.56 (1/177) [95% CI: 0.00;1.67]	5.88 (2/34) [95% CI: 0.00;13.8]	1.38 (6/434) [95% CI: 0.28;2.48]

N = Number of subjects.

% = Percentage of subjects with condition.

CI = Confidence Interval.

### Thromboembolic risk level of newly detected AF-patients

The CHA_2_DS_2_-VASc stroke prediction risk score was calculated for all patients, including those with an irregular pulse (Table 7). The calculated stroke risk in patients detected with AF should guide the GP in relation to risk management including anticoagulant treatment initiation. Interpretation of stroke risk and initiation of preventive actions was by that left at the discretion of the investigators as this was a non-intervention study.

**Table 7:** CHA_2_DS_2_-VASc score (Specialist verified cases (N = 10)).

Age group	65–74 (N = 480)	75–84 (N = 372)	≥85 (N = 118)	Total (N = 970)
- Mean score (SD)	2.5 (1.0)	3.6 (1.0)	3.9 (1.1)	3.1 (1.2)
**Irregular pulse**	21	39	27	87
- Mean score (SD)	2.4 (1.3)	3.6 (1.1)	3.8 (1.1)	3.4 (1.3)
**Atrial fibrillation**	4	2	4	10
- Mean score (SD)	3.8 (1.7)	3.0 (0.0)	3.0 (0.8)	3.3 (1.2)

N = Number of subjects.

% = Percentage of subjects with condition.

SD = Standard Deviation

For all patient groups the CHA_2_DS_2_-VASc score was predictably high and would prompt preventive stroke measures if AF was present. For the different age groups, all screened patients, except those diagnosed with AF, showed a gradual increase in the CHA_2_DS_2_-VASc score mostly driven by age as the most predominant discriminator. This finding was also true for patients detected with an irregular pulse, but could not be found for patients characterized as having AF, being close to the average of 3.3 for all age groups.

## Discussion

In the present study, we attempted from a routine daily clinical practice to conduct a real life examination of the ESC and the Danish Society of Cardiology guidelines recommendations adopting opportunistic screening of irregular pulse in a primary care setting to detect new AF patients. Of interest, we did detect patients with irregular pulse and ECG verified indication of AF by opportunistic screening–patients that were previously undetected and undiagnosed as documented in their medical records. The prevalence of irregular pulse was age dependent, with almost one in four patients above 85 years of age presenting with this potential marker of AF. The prevalence of ECG detected AF in the primary care setting also points at patients by increasing age as targets for this screening recommendation with one out of thirty screened being categorized with AF. The results found in terms of cases of irregular pulse as well as those with indication of AF were in line with the previous finding by opportunistic screening in Fitzmaurice et al. [12].

Compared to routine practice (normal clinical case finding) the study by Fitzmaurice et al. [12] found both *systematic screening* (by postal invitation to have an ECG) and *opportunistic screening* (pulse palpation at the GP setting when appearing in the clinic and ECG follow-up in case of irregular pulse) of people over the age of 65 years to be equally effective for the detection of AF (OR 1.57; 95% CI: 1.08; 2.26 (systematic screening) and OR 1.58; 95% CI: 1.10; 2.29 (opportunistic screening)) resulting in a detection rate of patients with unknown AF of 2.2% and 1.6%, respectively [3]. However, by opportunistic screening (167; 95% CI: 92 to 806) slightly fewer persons had to be screened to detect one additional case than during systematic screening (172; 95% CI: 94 to 927) [16]. In comparison to the present study it has, however, to be remembered that the study by Fitzmaurice et al. [12] has a 12 month study period compared to 3 months in the present study. Besides the study by Fitzmaurice et al. [12] and a study by Morgan et al. [17], other studies published concerning screening of AF have not focused on opportunistic screening of AF, but only on single time point systematic screening, as identified by a comprehensive review by Lowres et al. covering 30 studies representing nine countries (n = 122,571, mean age 64 years, 54% male) [32]. ECG was used as the single screening method in 25 of these studies. 12 studies, including Fitzmaurice et al. [12] and Morgan et al. [17], investigated screening in the GP or outpatient clinic setting with the majority of the studies focusing on patients’ ≥65 years [12,17–27]. The prevalence of AF in those ≥65 years across all studies was 4.4% (95% CI: 4.1–4.6), and the overall incidence of previously unknown AF (detection rate) in those ≥65 years was 1.4% (95% CI: 1.2–1.6) [32].

### Representativeness

The age distribution of the patients included opportunistically in our study was, overall, rather close to follow the age distribution of the Danish population [31], especially, for the group of the oldest patients (≥85 age). However, for the two other younger age groups the share of patients in the study was lower compared to their age-based share in the population (65–74: 49% (study) versus 59% and 75–84: 29% (study) versus 38%). These lower shares of patients screened in these age groups may explain the lower share of AF-patients found in the study for these groups. Compared with the present study a similar age distribution of the opportunistically screened patients was found in the opportunistic screening study arm in the study by Fitzmaurice et al. [12].

The highest number of clinics that were either not able to recruit patients to the study or had a rather low number of patients included in the study was among the clinics randomly allocated the ≥85 age group for their opportunistic screening activity. This could be caused by a lower proportion of very elderly in the general population, or that older patients are to a higher extent not visiting their GP, but are visited by their GP in their home, in nursing home or other remote care settings.

### Screening of AF in the GP setting

First of all the study demonstrated the importance of collaboration between the primary care and the specialist settings in terms of the final confirmation of the diagnosis of AF. This was due to the three (23%) misclassified AF patients following ECG interpretation in primary care and disqualified by consecutive specialist validation. However, since primary care, in their role as gatekeepers filter the vast majority of patients at risk of having AF they do have an important role in early screening and detection of AF, and in collaboration with skilled specialists to draw up decisions on how best to manage the AF patients, including long-term follow up, most often to be continued by primary care. This important role of the primary care setting is expected to even increase in the future.

The opportunistic screening carried out in the study was nurse-assisted under supervision and responsibility of the GP. Overall this way of handling opportunistic screening seemed to function well in the study which makes sense as the clinic nurse has a close contact with many patients during a day with the possibility for having a focus upon specific investigations such as pulse palpation of relevant patient categories. Similarly did Tagger et al. in a UK survey of 418 health care professionals conclude that opportunistic screening in the primary care setting is feasible and that there are adequate access to resources for AF screening in this setting [33]. Based on the survey results they supported a role for non-GP (e.g. nurse-assisted) involvement in future AF screening.

As supported in the UK setting by Tagger et al. [33] the same would seem reasonable to assume in the routine daily clinical practice in primary care in Denmark, because almost all patients suffering from a chronic disease (e.g. hypertension, diabetes, COPD) are by contract between Danish Regions and the general practice sector seen by their GP at least once a year. During these consultations a major fraction of asymptomatic cases of AF could be detected, as most of these clinical examinations will include investigations like pulse palpation, blood tests, and/or ECG. A major part of the remainder of the patients are also seen by their GP for any reason at least once a year, where a substantial part of asymptomatic cases of AF can be argued to be found as well. However, bearing in mind that this non-systematic screening is de facto already carried out with the examinations presented above, our finding in the present study of an overall specialist verified detection rate of a further 1.03% (10/970) of all subjects screened to be suffering from AF supports the AF guideline recommendation on continued and increased focus on opportunistic screening of AF in the primary care setting of the age groups being in focus in the present study. This is also supported by an expected annual incidence rate of new AF of 1% or higher among Danish citizens aged 65 years and older. Therefore, opportunistic screening in routine daily clinical practice will potentially shorten diagnostic lag time among subjects with ECG detectable AF, and therefore potentially prevent development of AF associated stroke, peripheral arterial thromboembolism, heart failure and death.

### AF screening and outcome

Screening for AF (opportunistic screening in particular), employing different kinds of set-ups and screening-modes available, including variable intensity among different types of screening, is recommended in guidelines [1,2,11,16]. There is no final proof yet that screening for AF followed by initiation of risk balanced oral anticoagulant treatment, translates into a better outcome. The currently ongoing Swedish STROKESTOP Study is, however, expected to report outcomes data from a randomized systematic AF screening study addressing this particular issue in the near future [34].

### Study limitations

We have no data on the detection rate of AF among GPs not participating in the present study. The opportunistic screening option in the present study was typically only tested for a period of few weeks up to a month in the participating clinics. Having performed the activity for a longer time period may have made it a routine to a higher degree and with more skills and experiences that could have improved the screening result further, including the ECG examination by the GPs. This is, however, something that has to be tested in the clinics, if opportunistic screening is to be rolled-out in the primary care setting on a larger scale. The present study has shown that opportunistic screening in the primary care setting is feasible.

In terms of the patients they were not randomized to opportunistic screening or no-screening or other screening types at the clinic level, as one would do in a randomized controlled clinical trial, e.g. such as Fitzmaurice et al. [12]. This kind of design was, however, not applied in the present study as the aim was to examine guideline recommendations based in the real-life setting in routine daily clinical practice. Furthermore, randomization at the patient level is not allowed in a non-interventional study design, because the randomization of patients itself changes the daily routine practice carried out for these patients. However, the consequence of this design with no randomization at the patient level and no control group is that the study is descriptive and explorative, and not testing hypothesis. This may limit the conclusions made from the study.

Patients were supposed to be included opportunistically in the study on a consecutive basis to avoid any pre-selection bias of study subjects. The fact that most clinics did reach the number of patients agreed within a relatively short study period indicates that the study was carried out on a consecutive basis. However, there was no system available to control for this, and thus we cannot exclude that some patients were not asked by the clinics to participate.

Due to this one arm design in routine daily clinical practice with patients entering consecutively without invitation analyses of non-participation were not made. Furthermore, data collection among non-participants was beyond the informed consent provided by patients. The lack of analysis of non-participation rates was though found to be less critical due to the routine daily clinical practice setting and with the expectation that the majority of patients entering consecutively and asked for participation accepted this.

Finally, at the clinic level, though, all clinics participating were cluster randomized to one of the three age groups without taking into account what age-span the patients in the specific clinic had. The consequence of this might have been that some of the clinics randomly selected for the ≥85 age groups were clinics with few of these patients in their patient pool relative to other age groups. This was also the overall reason given by these clinics to their lack of recruitment of ≥85 years aged patients that they had a limited number of “available to clinic” patients in this age-segment. A further reason was that the few ≥85 patients they actually had in the clinic were not visiting the clinic for any reason during the screening period.

### Extrapolation of results

There are roughly 3,600 general practitioners in Denmark. The present study enrolled active participation of 49 general practitioners and their clinic nurse who included 970 patients in a shorter screening period. This resulted in 10 patients with a specialist confirmed AF diagnosis. With at least 80% of the adult population seen by their GP in Denmark annually then this study documented the feasibility of having patients being detected and diagnosed with AF in the primary care setting.

## Conclusions

In support of the ESC guidelines and the guidelines endorsed by the Danish Society of Cardiology recommending opportunistic screening for AF, the present study has shown that nurse-assisted opportunistic screening in the primary care setting in Denmark is both feasible and leads to detection of patients with AF. From a public health perspective with the expected future growth in the prevalence of AF patients in Denmark and elsewhere, then continued and repeated pulse palpation of 65+ years of age patients visiting their GP for whatever reason, will result in detection and proper treatment of AF-patients. Furthermore, even though the primary care setting will play an increasingly important role in AF detection, diagnosis, treatment and follow-up in the future, the present study has also shown that this should be done in close collaboration with the AF-specialists, e.g. cardiologists. Finally, more focus and research on screening procedures will also be needed for the oldest and highest risk patients, as they might be less within reach in the GP-setting.

## Supporting information

S1 Table Pulse measurement and ECG results at the GP clinic—All data. (DOCX)

S2 Table CHA2DS2-VASc score (Specialist verified cases (N = 10))–All data. (DOCX)
