# Supplementary material for: Opportunistic screening for atrial fibrillation in a real-life setting in general practice in Denmark—The Atrial Fibrillation Found On Routine Detection (AFFORD) non-interventional study
Source: PLoS One. 2017 Nov 13;12(11):e0188086. doi: 10.1371/journal.pone.0188086 (PMC5683635; doi:10.1371/journal.pone.0188086)
Supplement: S1 Table — (DOCX) [file pone.0188086.s001.docx]

## Table S1. Pulse measurement and ECG results at the GP clinic – all data

|  | | 65-74 | 75-84 | 85+ | Total |
| --- | --- | --- | --- | --- | --- |
| Pulse measurement by arterial palpation at wrist | N | 480 | 372 | 118 | 970 |
|  | Mean (SD) | 72.5 ( 11.5) | 72.7 ( 11.3) | 73.9 ( 10.9) | 72.8 ( 11.3) |
|  | Median | 72.0 | 72.0 | 76.0 | 72.0 |
|  | 95 % CI lower - upper | 71.5 - 73.6 | 71.6 - 73.9 | 72.0 - 75.9 | 72.1 - 73.5 |
|  | Min - Max | 47.0 - 130.0 | 48.0 - 106.0 | 50.0 - 96.0 | 47.0 - 130.0 |
|  | Missing | 0 ( 0.00) | 0 ( 0.00) | 0 ( 0.00) | 0 ( 0.00) |
| Pulse measurement findings | N | 480 | 372 | 118 | 970 |
|  | Irregular | 21 ( 4.4) | 39 ( 10.5) | 27 ( 22.9) | 87 ( 9.0) |
|  | Regular | 459 ( 95.6) | 333 ( 89.5) | 91 ( 77.1) | 883 ( 91.0) |
| Atrial fibrillation / EKG | N (irregluar pulse) | 21 | 39 | 27 | 87 |
|  | Yes | 5 ( 23.8) | 4 ( 10.3) | 4 ( 14.8) | 13 ( 14.9) |
|  | No | 16 ( 76.2) | 35 ( 89.7) | 23 ( 85.2) | 74 ( 85.1) |
| Table notes:  N = Number of subjects % = Percent of subjects randomized FAS: All included subjects | | | | | |
