# Supplementary material for: Opportunistic screening for atrial fibrillation in a real-life setting in general practice in Denmark—The Atrial Fibrillation Found On Routine Detection (AFFORD) non-interventional study
Source: PLoS One. 2017 Nov 13;12(11):e0188086. doi: 10.1371/journal.pone.0188086 (PMC5683635; doi:10.1371/journal.pone.0188086)
Supplement: S2 Table — (DOCX) [file pone.0188086.s002.docx]

| Table S2. CHA2DS2-VASc score (Specialist verified cases (N=10)) – all data | | | | | |
| --- | --- | --- | --- | --- | --- |
|  | | 65-74 | 75-84 | 85+ | Total |
| All patients | N | 480 | 372 | 118 | 970 |
|  | Mean (SD) | 2.5 ( 1.0) | 3.6 ( 1.0) | 3.9 ( 1.1) | 3.1 ( 1.2) |
|  | Median | 2.0 | 4.0 | 4.0 | 3.0 |
|  | 95 % CI lower - upper | 2.4 - 2.6 | 3.5 - 3.7 | 3.7 - 4.1 | 3.0 - 3.2 |
|  | Min - Max | 1.0 - 6.0 | 2.0 - 7.0 | 2.0 - 7.0 | 1.0 - 7.0 |
|  | Missing | 0 ( 0.00) | 0 ( 0.00) | 0 ( 0.00) | 0 ( 0.00) |
| AF | N | 4 | 2 | 4 | 10 |
|  | Mean (SD) | 3.8 ( 1.7) | 3.0 ( 0.0) | 3.0 ( 0.8) | 3.3 ( 1.2) |
|  | Median | 3.5 | 3.0 | 3.0 | 3.0 |
|  | 95 % CI lower - upper | 1.0 - 6.5 |  | 1.7 - 4.3 | 2.5 - 4.1 |
|  | Min - Max | 2.0 - 6.0 | 3.0 - 3.0 | 2.0 - 4.0 | 2.0 - 6.0 |
|  | Missing | 0 ( 0.00) | 0 ( 0.00) | 0 ( 0.00) | 0 ( 0.00) |
| No AF | N | 17 | 37 | 23 | 77 |
|  | Mean (SD) | 2.1 ( 1.0) | 3.6 ( 1.1) | 4.0 ( 1.1) | 3.4 ( 1.3) |
|  | Median | 2.0 | 4.0 | 4.0 | 3.0 |
|  | 95 % CI lower - upper | 1.6 - 2.6 | 3.2 - 4.0 | 3.5 - 4.4 | 3.1 - 3.7 |
|  | Min - Max | 1.0 - 5.0 | 2.0 - 7.0 | 2.0 - 6.0 | 1.0 - 7.0 |
|  | Missing | 0 ( 0.00) | 0 ( 0.00) | 0 ( 0.00) | 0 ( 0.00) |
| Irregular pulse | N | 21 | 39 | 27 | 87 |
|  | Mean (SD) | 2.4 ( 1.3) | 3.6 ( 1.1) | 3.8 ( 1.1) | 3.4 ( 1.3) |
|  | Median | 2.0 | 4.0 | 4.0 | 3.0 |
|  | 95 % CI lower - upper | 1.8 - 3.0 | 3.2 - 3.9 | 3.4 - 4.2 | 3.1 - 3.6 |
|  | Min - Max | 1.0 - 6.0 | 2.0 - 7.0 | 2.0 - 6.0 | 1.0 - 7.0 |
|  | Missing | 0 ( 0.00) | 0 ( 0.00) | 0 ( 0.00) | 0 ( 0.00) |
| Table notes:  N = Number of subjects % = Percent of subjects randomized FAS: All included subjects For subjetID 2110191261, 2110191277, 2110191859 AF is set to No | | | | | |
